# Cholesterol-Peptide Hybrids to Form Liposome-Like Vesicles for Gene Delivery

**DOI:** 10.1371/journal.pone.0054460

**Published:** 2013-01-30

**Authors:** Qiong Tang, Bin Cao, Haiyan Wu, Gang Cheng

**Affiliations:** 1 Department of Chemical and Biomolecular Engineering, University of Akron, Akron, Ohio, United States of America; 2 Department of Integrative Medical Sciences, Northeast Ohio Medical University, Rootstown, Ohio, United States of America; The Ohio State University, United States of America

## Abstract

In this paper, four amphiphilic cholesterol-peptide conjugates (Ch-R5H5, Ch-R3H3, Ch-R5 and Ch-R5) were designed and synthesized, and their properties in gene delivery were evaluated in vitro with an aim of developing more efficient gene delivery carriers. These amphiphilic cholesterol-peptide conjugates are composed of hydrophobic cholesterol and positively charged peptides. They were able to self-assemble into micelles at low concentrations and their critical micelle concentrations in phosphate buffered saline (pH 7.4) are ≤85 µg/mL. Amphiphilic cholesterol-peptide conjugates condensed DNA more efficiently than a hydrophilic cationic oligoarginine (R10) peptide with no hydrophobic segment. Their transfection efficiencies were at least two orders of magnitude greater than that of R10 peptide in HEK-293 cells. Moreover, the introduction of histidine residues in cholesterol-peptide conjugates led to higher gene expression efficiency compared with cholesterol-peptides without histidine (Ch-R5 and Ch-R3), and the luciferase expression level was comparable or even higher than that induced by PEI at its optimal N/P ratio. In particular, Ch-R5H5 condensed DNA into smaller nanoparticles than Ch-R3H3 at higher N/P ratios, and the minimum size of Ch-R5H5/DNA complexes was 180 nm with zeta potential of 23 mV, achieved at the N/P ratio of 30. This liposome-like vesicle may be a promising gene delivery carrier for intravenous therapy.

## Introduction

Gene therapy has become a major research focus in recent years since it covers a broad range of applications in gene silencing or replacement for genetic or acquired diseases [1], [2]. To achieve this goal, various delivery systems have been developed. Generally, they can be classified into two categories: viral vectors and non-viral vectors [3], [4]. Viral vectors exhibit a high efficiency in DNA delivery *in vitro*, but their clinical application is dramatically limited due to immunogenicity and toxicity [2], [5], [6]. Non-viral delivery systems have shown considerable clinical potential, and a large amount of effort has been made in this field. Currently, the majority of non-viral delivery systems are based on synthetic materials, including cationic lipids, polymers, and dendrimers [7]–[9], and some of them could induce high gene transfection [10]. However, many of these systems are of the low biodegradability and poor biocompatibility.

With some advantages over synthetic materials, natural materials such as cationic peptides [11] and polysaccharides [12] have been proposed as gene delivery vectors. In particular, peptide-based carriers are amenable to rational design and development since amino acids with diverse properties provide us the freedom for developing multifunctional drug carriers with the desired functions. For example, cationic peptides have been used to condense negatively charged therapeutic DNA or RNA, and deliver them to cells [13]–[16]. Histidine residue is introduced into peptides to help DNA escape from endosome and thus to improve transfection efficiency [17], [18]. In addition, it is reported that adding hydrophobic compounds, such as cholesterol [19], lipids [20], and peptides [21], into peptide vectors could induce the formation of micelle-like nanoparticles and thus increase the local cationic charge density in the solution, which allows for better complexation of DNA. However, there are two major obstacles for the further development of natural polymer-based gene carriers. Firstly all of the reported gene drug vectors provide only part of required functions that an efficient drug delivery system needs to have. Secondly the gene transfection efficiency of natural polymer is low in general. Our research effort is focused on developing biodegradable, effective and integrated gene delivery system. Due to the cost and complex structure of long peptides (>15 amino acids), we are particularly interested in small peptides (<15 amino acids) for gene delivery. However the transfection efficiency of small peptides is usually low, since they cannot efficiently condense DNA [22], [23]. A better understanding on the structure-function correlation of cationic peptide will help us to design more effective gene delivery systems.

In this paper, we designed and synthesized four small amphiphilic cholesterol-peptides conjugates (Ch-R5H5, Ch-R3H3, Ch-R5 and Ch-R3). In our previous study, we developed a peptide-based gene delivery vector, K_12_H_6_V_8_, in which lysine was used as a cationic block for DNA binding, histidine was used to help endosomal escape and valine was used to constitute micelle-forming hydrophobic block [24]. Here, to enhance the DNA binding ability, a more hydrophobic block (cholesterol) was used instead of the valine residue, and the lysine block was replaced by an arginine block to stabilize the core/shell structure. The properties of small amphiphilic cholesterol-peptide conjugates to condense DNA were studied. The utility of the cationic amphiphilic cholesterol-peptides for gene delivery was investigated in a human embryonic kidney cell line HEK-293 and a human breast cancer cell line MCF-7.

## Materials and Methods

### Materials

Tetrahydrofuran **(**THF), dimethyl sulfoxide (DMSO), triethylamine (TEA), acryloyl chloride, methanol, Tris-Borate-EDTA (TBE) buffer, phosphate-buffered saline (PBS), and polyethylenimine (PEI, branched, Mw 25 kDa) were purchased from Sigma-Aldrich (St Louis, MO, USA). Cholesterol (95%) and pyrene (98%) were purchased from Alfa Aesar (Ward Hill, MA, USA). The peptides, NH_2_-RRRRRHHHHHC-COOH (R5H5), NH_2_-RRRHHHC-COOH (R3H3), NH_2_-RRRRRC-COOH (R5), NH_2_-RRRC-COOH (R3), NH_2_-RRRRRRRRRR-COOH (R10) were designed by us and synthesized by GenScript (Piscataway, NJ, USA) at >95% purity. Agarose, 3-(4,5-Dimethylthiazol-2-yl)-2,5-Diphenyltetrazolium Bromide (MTT), Dulbecco’s Modified Eagle Medium (DMEM), fetal bovine serum (FBS), penicillin, streptomycin, non-essential amino acids, sodium pyruvate, L-glutamine, Trypsin-EDTA and Vybrant® MTT cell proliferation assay kit were all purchased from Life Technologies (Carlsbad, CA, USA). Ethidium bromide and nuclease-free water were purchased from EMD Millipore (Billerica, MA, USA). DNA loading dye was purchased from New England Biolabs (Ipswich, MA, USA). Glo lysis buffer and luciferase assay system were purchased from Promega (Madison, WI, USA). BCA protein assay kit was purchased from Thermo Scientific (Waltham, MA, USA). Plasmid DNA encoding a 5.2 kb firefly luciferase (pCMV-luc) was purchased from Elim Biopharmaceuticals (Hayward, CA, USA), amplified in *Escherichia coli* strain NovaBlue from EMD Millipore and purified with a plasmid maxi kit supplied by Omega Bio-Tek (Norcross, GA, USA). MCF-7 and HEK-293 cell lines were purchased from ATCC (St. Cloud, MN, USA).

### Synthesis of Cholesterol-peptide Conjugates

Cholesterol-acrylate was synthesized following a published procedure [25]. To a solution of cholesterol (4 g, 10.3 mmol) and TEA (4.3 mL, 31 mmol) in 50 mL of anhydrous THF, acryloyl chloride (1.3 mL, 15.6 mmol) was mixed with 5 mL of anhydrous THF and added dropwise at 0°C under a positive nitrogen flow. Then the mixture was removed from the ice-bath, warmed up to room temperature, and kept stirring overnight. After the removal of the solvent by rotary evaporator, the crude solid was redissolved in 50 mL of CH_2_Cl_2_, and washed with the following solutions: deionized (DI) water, HCl (0.5 M), DI water, NaHCO_3_ (1 M), DI water, and brine. The product in CH_2_Cl_2_ was dried over anhydrous MgSO_4_ overnight, and purified by silica gel column chromatography (CH_2_Cl_2_/Hexane 1/1 v/v) after the removal of the solvent by rotary evaporator. Pure product was obtained as a white solid (yield: 35%). The structure was confirmed with both ^1^H and ^13^C NMR. ^1^H NMR (300 MHz CDCl_3_) δ 6.39 (dd, 1H), 6.11 (dd, 1H), 5.80 (dd, 1H), 5.40 (d, 1H), 4.70 (m, 1H), 2.37 (d, 2H), 0.8–2.1 (m, 38H), 0.69 (s, 3H). ^13^C NMR (300 MHz CDCl_3_) δ 165.85, 139.85, 130.40, 129.29, 122.94, 74.35, 56.93, 56.39, 50.28, 42.55, 39.98, 39.75, 38.34, 37.23, 36.84, 36.42, 36.02, 32.11, 28.45, 28.26, 28.00, 24.51, 24.07, 23.03, 22.78, 21.27, 19.55, 18.95, 12.08. Cholesterol-acrylate and peptide (R5H5, R3H3, R5, or R3) conjugation was carried out in a mixture of DMSO and THF at the presence of TEA. The mixture was stirred at 37°C for 2 days under nitrogen protection. Pure product was obtained by dialysis against deionized water using a molecular weight cut-off of 100–500 D dialysis tube from Spectrum Laboratories (Rancho Dominguez, CA, USA) for one day, followed by freeze-drying.

### Determination of the Critical Micelle Concentration (CMC) of Cholesterol-peptide Conjugates

A series of cholesterol-peptide solutions containing 24 µM pyrene were prepared following the procedure in Dominguez’s paper [26]. Briefly, 100 µL of pyrene solution (0.025 mg/mL in methanol) was added to a series of vials and the solvent was evaporated, then the appropriate volumes of cholesterol-peptide solutions (250 µg/mL in PBS) and PBS buffer were added to obtain cholesterol-peptide concentrations ranging from 1 to 250 µg/mL at a total final volume of 500 µL. The mixed solutions were stirred at room temperature overnight, and then excited at 334 nm and the emission spectra were recorded from 360 to 410 nm using a PerkinElmer LS45 fluorescence spectrometer (Waltham, MA, USA). The intensity ratios of I_384_ to I_373_, corresponding to the third and first vertical peaks respectively, were plotted as a function of cholesterol-peptide concentration. The CMC value was obtained from the intersection of the tangents to the two linear portions of the graph.

### Preparation of Cholesterol-peptide/DNA Complexes

The cholesterol-peptides were dissolved in PBS buffer to make a stock solution at 10 mg/mL. The DNA was prepared at a concentration of 1 mg/mL in nuclease-free water. The cholesterol-peptide/DNA complexes were formed by mixing equal volume of cholesterol-peptide and DNA solutions at various N/P ratios, and then incubated at room temperature for 20 minutes to allow for complete electrostatic interaction between peptide and DNA. Here, the N/P ratio is a molar ratio of arginine residue (N) in the peptide to phosphate group (P) in the DNA molecule. The phosphate content of the DNA molecules was estimated based on the assumed 330 g/mol of the average molecular weight of the nucleotides, while the nitrogen content of the peptide molecule was estimated based on the number of arginine residues in each peptide molecule.

### Size and Zeta-potential Measurements

Peptide/DNA complexes were prepared as described above at different N/P ratios. The size and zeta-potential of the cholesterol-peptide/DNA complexes were measured by a Malvern Nano-ZS Zetasizer (UK). The size measurements were performed in disposable sizing cuvettes at a laser wavelength of 633 nm and a scattering angle of 173°, while the zeta-potential measurements were performed in disposable zeta-potential cells. Before the measurement, the cholesterol-peptide complexes were diluted 10 times with PBS, and each measurement was repeated for 3 runs per sample at 25°C.

### Gel Retardation Assay

The cholesterol-peptide/DNA complex solutions were prepared at N/P ratios from 0 to 15. An aliquot (5 µL, 0.5 µg DNA) of each solution was mixed with loading dye and loaded to an agarose gel (0.8%). The loaded gel was exposed to 100 V for 40 min in 0.5×TBE buffer, and was stained in ethidium bromide (0.5 µg/mL) for 30 minutes and destained with water for 15 minutes. Then the gel was visualized and documented using a UVP BioDoc-It imaging system (Upland, CA, USA).

### Cell Culture

HEK-293 and MCF-7 cells were cultured in DMEM (high glucose) under a 5% CO_2_ environment at 37°C. The medium was supplemented with 10% fetal bovine serum, non-essential amino acids, sodium pyruvate and penicillin-streptomycin. HEK-293 and MCF-7 cells were both seeded at a density of 4×10^5^ cells/mL in 24-well plate (0.5 mL/well) or 96-well plate (0.1 mL/well), and incubated for 24 hours to reach 80–90% confluence before treatment.

### In vitro Gene Transfection and Expression

To evaluate the efficiency of cholesterol-peptide conjugates for gene drug delivery, a plasmid containing a firefly luciferase reporter gene from *Photinus pyralis* was used as a model gene drug to form cholesterol-peptide/DNA complexes. After the complexes were prepared as described above, an equal volume of fresh medium was mixed with the complexes, and the solutions were incubated at room temperature for 10 minutes before transferred to a 24-well plate seeded with cells. For each well, old medium was replaced with 400 µL of fresh growth medium and 100 µL of the complex solution (containing 2.5 µg DNA). After 4 hours of incubation, the cells were washed with PBS and incubated with fresh medium for 68 hours. Then cells were washed with PBS and 0.2 mL of 1× Glo lysis buffer was added to each well to lyse the cells. After being incubated at room temperature for 15 minutes and subjected to two cycles of freezing (−80°C for 30 min) and thawing (room temperature), the cell lysates were centrifuged at 14,000 rpm at 4°C for 15 min to remove cell debris. The supernatant (20 µL) was transferred to a luminometer tube and mixed with 100 µL of luciferase assay reagent, then the luciferase activity was immediately measured for a 10-second read by a Berthold Lumat LB9507 luminometer (Germany). The relative light unit (RLU) value from the luminometer was normalized by the protein content in the supernatant which was determined by the BCA protein assay, and all the experiments were performed with 4 replicates.

### Cytotoxicity Assay

The cytotoxicity of cholesterol-peptide/DNA complexes was evaluated against HEK-293 and MCF-7 cell lines in 96-well plate, using Vybrant® MTT Cell Proliferation Assay. [27] After incubation for 24 hours, the medium in 96-well plate was replaced with 80 µL of fresh growth medium and 20 µL of the complex solution as prepared in the section of gene transfection and expression was added to each well. The cells were incubated at 37°C for 4 hours, then washed with PBS and incubated with fresh medium for 20 hours. The medium was then replaced with fresh one (100 µL) and 10 µL of MTT stock solution (5 mg/mL in PBS), and the cells were incubated for 4 hours at 37°C. Finally, the medium was removed and 150 µL of DMSO was added to each well to dissolve the purple formazan crystals. The absorbance was measured at 570 nm using a Tecan Infinite 200 microplate reader (Switzerland). The cytotoxicity test was performed in 8 replicates of each sample. The cells without any treatment were used as a control (100% cell viability), and the cell viability was expressed as a percentage of the control.

### Statistical Analysis

Data were analyzed using single-factor analysis of variance (ANOVA), and expressed as mean ± standard deviation from 4 to 8 replicates. The comparison among groups was performed using Student’s t-test. A *p* value less than 0.05 was considered to be statistically significant.

## Results and Discussion

**Figure 1:**
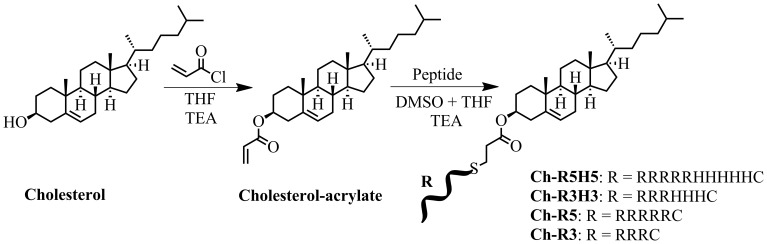
Synthesis of cholesterol-peptide conjugates.

Cholesterol was modified with acryloyl chloride to make cholesterol-acrylate and then conjugated to four peptides (R5H5, R3H3, R5 and R3) containing a C-terminal cysteine residue, respectively, through a very efficient thiol-acrylate Michael type reaction at slightly basic condition (Fig. 1) [28]. To avoid the steric hindrance on the conjugation and enhance the charge density, cysteine residue was put at the C-terminal of the peptide. This approach does not require the modification of the peptide and can be used to conjugate any peptide sequences with a terminal cysteine residue.

**Figure 2:**
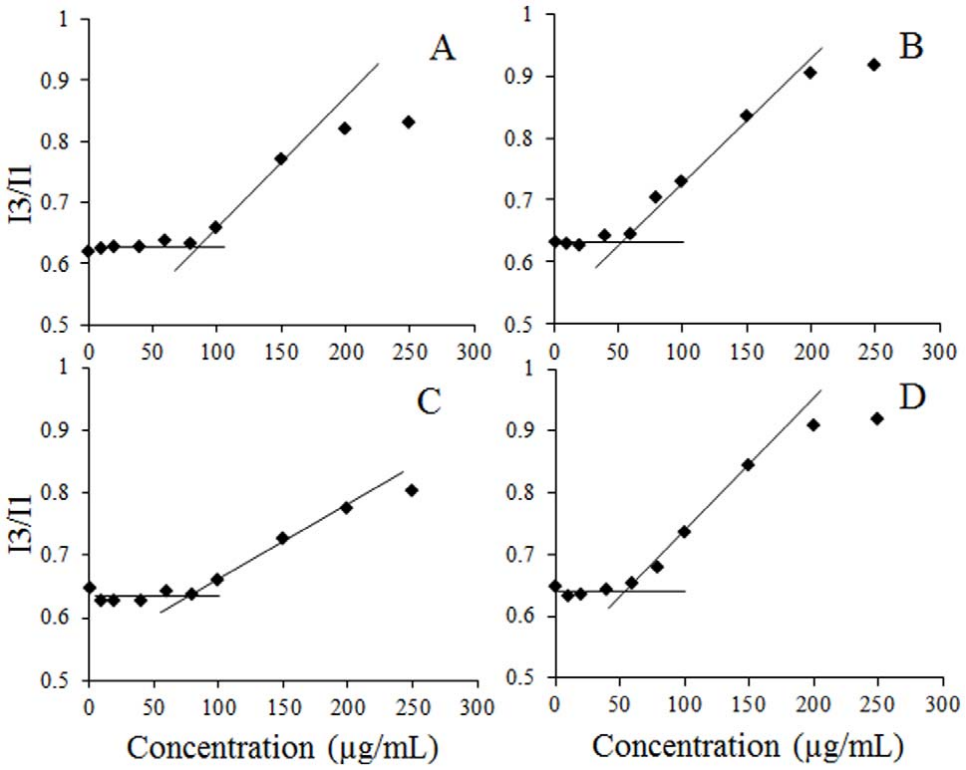
Critical micelle concentration of cholesterol-peptide hybrids in PBS at 25°C. Panel A. Ch-R5H5. Panel B. Ch-R3H3. Panel C. Ch-R5. Panel D. Ch-R3.

To study the aggregation behavior of cholesterol-peptides, the critical micelle concentration (CMC) was determined by fluorescence spectrometer using pyrene as a hydrophobic probe. Since pyrene preferentially solubilizes itself into the hydrophobic environment, the fluorescence intensity of pyrene shows a strong dependence on solvent environment. In the absence of micelles (below the CMC), pyrene disperses in the polar environment (water) and the ratio of fluorescence emission intensities of the third peak to the first peak (I_3_/I_1_) is low. Above the CMC, pyrene molecules insert into the hydrophobic domains of the core of the micelles, and the ratio of I_3_ to I_1_ increases. The ratio of I_3_ to I_1_ was plotted against the concentration of cholesterol-peptides, and the CMC was calculated by the interception of two straight lines (Fig. 2). The CMC values of the cholesterol-peptides in PBS buffer (pH 7.4) were determined to be ∼85 µg/mL for Ch-R5H5 and Ch-R5, and ∼50 µg/mL for Ch-R3H3 and Ch-R3. The CMC value of cholesterol-peptide conjugates with 5 arginine residues was higher than that with 3 arginine residues, while cholesterol-peptides containing the same amount of arginine amino acids achieved similar CMC values no matter it has a histidine block or not. At pH 7.4, arginine is positively charged while histidine is neutral. Hence, with the same amount of cholesterol moiety per conjugated peptide, Ch-R5H5 and Ch-R5 have a longer hydrophilic segment from the arginine block than Ch-R3H3 and Ch-R3 which needs to be counteracted by hydrophobic interaction to form micelles, leading to a higher CMC value [29].

Particle size is one of the most important factors that influence the efficiency of gene transfection *in vitro* and *in vivo*. As shown in Table 1, the size of Ch-R5H5/DNA complexes increased to its maximum (718 nm) at the N/P ratio of 5, and followed a decreasing trend as the N/P ratio increased from 10 to 30. Among all tested N/P ratios, the smallest complexes were formed at the N/P ratio of 30 (179 nm) for Ch-R5H5. Similar phenomena of charge ratio-dependent size change have been reported in the formation of DNA-liposome complexes. The main feature is the formation of large equilibrium clusters near the isoelectric point, due to the aggregation of intact polyion-coated vesicles. At increasing N/P ratio, the size of the clusters continuously increases, reaching a maximum at a well-defined value of this ratio, and then decreases (“reentrant” condensation) [30]. In our Ch-R5H5 system, the large equilibrium cluster formed at N/P 5 which is near the transition of surface charge from negative to positive (isoelectric point). For Ch-R3H3, Ch-R5 and Ch-R3, the sizes of these cholesterol-peptide/DNA complexes increased all the way as N/P ratio went up from 1 to 30, which did not follow the same trend as Ch-R5H5/DNA complexes. The difference may be explained by the shorter hydrophilic block in Ch-R3H3, Ch-R5 and Ch-R3, compared with that in Ch-R5H5. The underlying mechanism is not clear yet. A possible explanation for the increase in size as N/P ratio goes up is that at high N/P ratios some cholesterol molecules may radiate from the complex surfaces when arginine blocks have bonded to DNA via electrostatical interaction [21]. This may cause particle aggregation. In general, the shorter the hydrophilic cationic block was; the larger the complexes were formed at specific N/P ratios (Table 1). Compared with hydrophilic R_10_ peptide with no hydrophobic block, the cholesterol-peptide conjugates can pack DNA much more efficiently. Since introducing the hydrophobic cholesterol into hydrophilic peptides enables hybrid molecules self-assemble into liposome-like vesicles in aqueous solution, which interact stronger with DNA and thus form smaller particles [29]. The size result of Ch-peptide/DNA complexes indicates tuning the ratio of hydrophilic and hydrophobic blocks is important to obtain desired nanoparticles for gene drug delivery.

**Table 1:** Hydrodynamic size and zeta potential of Ch-R5H5/DNA, Ch-R3H3/DNA, Ch-R5/DNA, Ch-R3/DNA and R10/DNA complexes.

Carrier	N/P	Size (d.nm)	SD (±d.nm)	Zeta (mV)	SD (±mV)
Ch-R5H5	1	278	15	−15.7	1.5
	5	718	82	12.1	0.1
	10	367	66	17.2	1.3
	15	189	16	21.7	1.4
	20	233	27	21.4	1.0
	30	179	15	22.5	1.4
Ch-R3H3	1	246	16	−29.2	2.4
	5	505	54	15.1	3.7
	10	463	77	21.0	0.2
	15	604	62	20.9	1.6
	20	805	108	22.0	0.6
	30	793	53	22.7	0.7
Ch-R5	1	295	32	−27.5	2.5
	5	690	216	13.2	1.6
	10	863	196	18.5	0.4
	15	1327	96	18.0	1.0
	20	1682	50	19.7	0.5
	30	1618	188	19.6	1.0
Ch-R3	1	513	14	−34.7	2.6
	5	628	265	−18.1	2.8
	10	1134	327	17.2	0.4
	15	1344	170	18.8	0.8
	20	1531	230	18.6	1.1
	30	1755	51	17.7	0.9
R10	1	1121	100	−14.6	0.4
	5	4322	87	−2.4	0.1
	10	3881	121	0.6	0.1
	15	4636	95	6.0	0.5
	20	4634	38	6.6	0.8
	30	4597	41	7.0	1.4

Zeta potential of vector/DNA complexes also plays an important role in determining the gene transfection efficiency. A net positive charge of vector/DNA complex is preferred since it could interact with the negatively charged cell membrane and thus be internalized by cells. However, the high positive charge density of vector/DNA complexes might induce cellular toxicity [31]. As shown in Table 1, at the N/P ratio of 1, the zeta potential of all the cholesterol-peptide/DNA complexes was negative. As the N/P ratio was increased, the zeta potential of complexes increased as well, indicating more positively charged molecules (cholesterol-peptides) were condensed onto each DNA molecule. Above the N/P ratio of 10, the zeta potential did not change too much, and stayed around 23 mV for Ch-R5H5 and Ch-R3H3, 20 mV for Ch-R5 and 18 mV for Ch-R3, at the N/P ratio of 30. The results showed that the length of the hydrophilic block dramatically affects the condensation ability of the cholesterol-peptides. In addition, compared with cholesterol-peptide conjugates, R10 had a lower charge density and thus had a weaker interaction with DNA, indicated by much lower zeta-potential values of R10/DNA complexes (Table 1).

**Figure 3:**
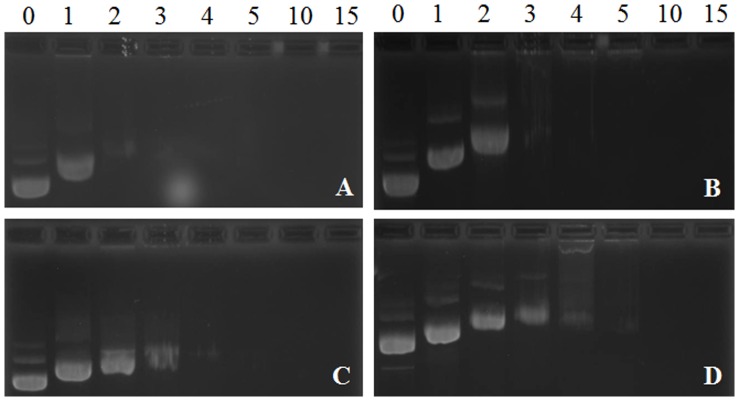
Electrophoretic mobility of DNA in cholesterol-peptide/DNA complexes at N/P ratio of 0, 1, 2, 3, 4, 5, 10 and 15 respectively. Panel A. Ch-R5H5/DNA complexes, Panel B. Ch-R3H3/DNA complexes. Panel C. Ch-R5/DNA complexes. Panel D. Ch-R3/DNA complexes.

The DNA binding ability of cholesterol-peptides was evaluated by an agarose gel retardation assay. The cholesterol-peptide/DNA complexes were formed at N/P ratios ranging from 0 to 15. As shown in Fig. 3, the complete retardation of DNA for four cholesterol-peptide conjugates was achieved below the N/P ratio of 10. The binding ability of the cholesterol-peptides followed a decreasing order as Ch-R5H5> Ch-R3H3> Ch-R5> Ch-R3. For Ch-R5H5, the complete retardation of DNA was achieved at the N/P ratio as low as 3. The results from the agarose gel retardation assay also agreed with the results of zeta-potential measurement.

**Figure 4:**
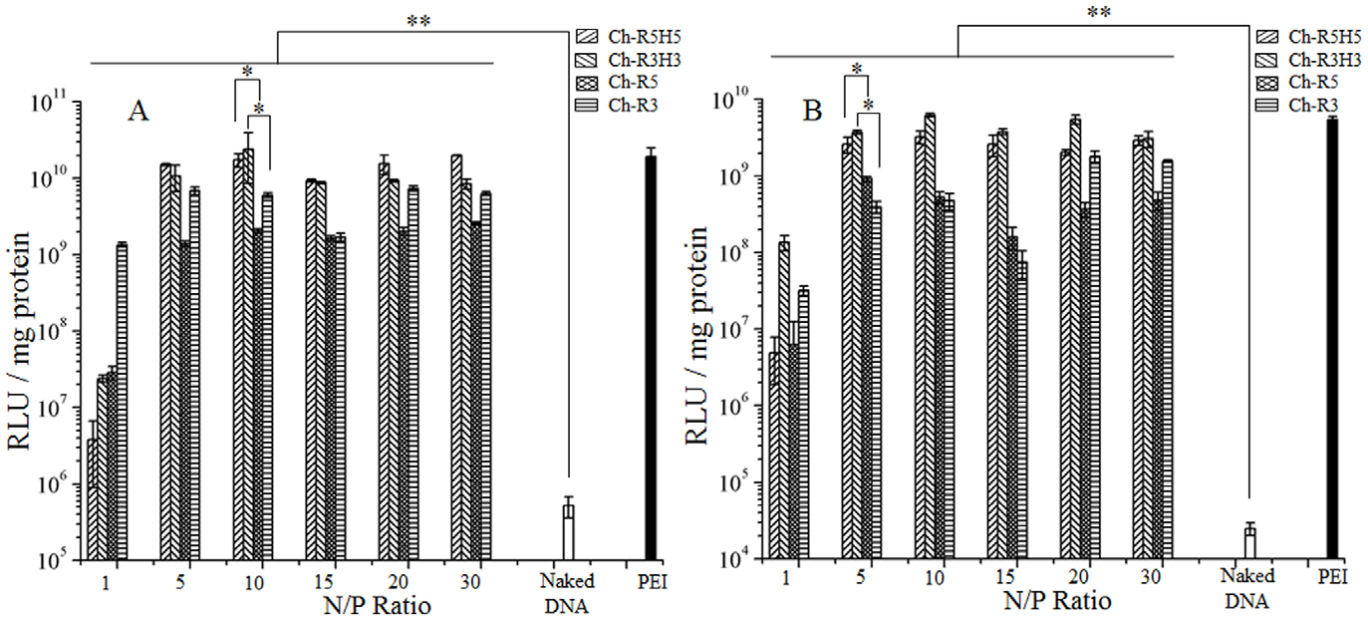
Luciferase expression levels induced by Ch-R5H5/DNA, Ch-R3H3/DNA, Ch-R5, and Ch-R3 complexes in mammalian cells. DNA and PEI/DNA complex at an N/P ratio of 10 are used as negative and positive controls respectively. Error bars represent standard deviation of 4 replicates. Panel A. HEK-293 cell line. Panel B. MCF-7 cell line.

The luciferase expression efficiency induced by four cholesterol-peptide/DNA complexes was studied at N/P ratios of 1, 5, 10, 15, 20 and 30 against HEK-293 and MCF-7 cell lines, and the results were compared with that induced by naked DNA and PEI/DNA at the N/P ratio of 10. As shown in Fig. 4, at the N/P ratio of 1, the average luciferase expression level of the 4 cholesterol-peptides was ∼300 and ∼10 times higher than that of the naked DNA in HEK-293 and MCF-7 cell lines respectively, but over 100 times lower than those at higher N/P ratios. It indicated that the cholesterol-peptides could facilitate the cellular internalization of DNA even when the surface charge of carrier/DNA complexes was negative. Above the N/P ratio of 1, all cholesterol-peptide conjugates were present as the form of micelles in the solutions of cholesterol-peptide/DNA complexes since the concentrations of the cholesterol-peptide conjugates were higher than their CMC values. At N/P ratios from 5 to 30, the luciferase expression induced by each cholesterol-peptide conjugate generally stayed at a similar level, which did not follow the trend as size or zeta-potential of cholesterol-peptide/DNA particles. Results indicated that larger particles (0.5∼1.7 µm) could also induce a relatively high luciferase expression. For example, at the N/P ratio of 20, the luciferase expression level induced by Ch-R3H3/DNA complexes (800 nm) was 2.7 times higher than that yielded by the complexes of Ch-R5H5/DNA at 300 nm in MCF-7 cell line (Fig. 4B). For Ch-R3 and Ch-R5, peak luciferase expression levels were only slightly lower than that of PEI in spite of the large particle size (∼1.7 µm). It has been reported that large particles could induce high transfection efficiency in vitro, partially because of a sedimentation effect [21]. Further research is necessary to understand the underlying mechanism.

As mentioned in the previous section, the formation of micelles increased the cationic charge density of carriers in the solution, which led to a stronger DNA binding ability of cholesterol-peptides, and thus induced a much higher gene expression than that did at the N/P ratio of 1 where micelles were not formed. Meanwhile, the effect of the histidine block on gene expression efficiency was also studied. The cholesterol-peptide conjugates with histidine residues (Ch-R5H5 and Ch-R3H3) induced a significantly higher luciferase expression than the cholesterol-peptides without histidine, respectively. In HEK-293 cells, the highest increase induced by Ch-R5H5 and Ch-R3H3 were achieved at N/P ratios of 10 (11 fold) and 5 (5 fold), respectively; in MCF7 cells, the highest expression increase, 16 fold and 51 fold, were achieved for Ch-R5H5 and Ch-R3H3 respectively at the N/P ratio of 5. This result confirmed that adding histidine in a DNA vector would induce a higher gene expression since the buffering capacity of histidine facilitates DNA to escape from endosomes, according to the proton sponge hypothesis [18]. Our results demonstrated that the peptide-based amphiphilic DNA carrier is more effective than hydrophilic cationic peptide, oligoarginine (R10). In HEK-293 cells, the gene expression induced by Ch-R5H5 is at least two orders of magnitude higher than that of the R10 peptide with no hydrophobic segment at N/P ratios of 5 to 30. In comparison with the peptide-based gene vector K_12_H_6_V_8_ from our previous study, Ch-R5H5 induced 3000-fold higher luciferase expression [24], indicating cholesterol is a better choice than valine as a hydrophobic block. Overall, by adding functional blocks (cholesterol and histidine) into the gene vectors, the gene expression induced by Ch-R5H5/R3H3 had been increased greatly which was comparable or even higher than that yielded by PEI at its optimal N/P ratio of 10.

**Figure 5:**
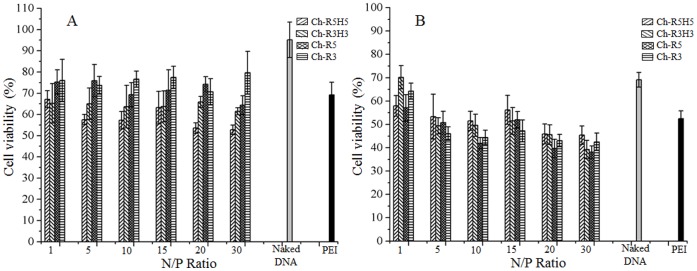
Cell viability of mammalian cells after incubation with Ch-R5H5-DNA, Ch-R3H3-DNA, Ch-R5, and Ch-R3 complexes at different N/P ratios compared to DNA and PEI/DNA complex at an N/P ratio of 10. Error bars represent standard deviation of 8 replicates. Panel A. HEK-293 cell line. Panel B. MCF-7 cell line.

To evaluate the potential application of the cholesterol-peptides as gene drug carriers, cytotoxicity of cholesterol-peptide/DNA complexes was determined against HEK-293 and MCF-7 cell lines. As shown in Fig. 5, the cell viability depended on cell type and N/P ratio. In HEK-293 cells, there was no clear trend observed in cytotoxicity among Ch-R3H3, Ch-R5 and Ch-R3, as the N/P ratio was increased, but a slightly decreasing trend was found for Ch-R5H5. On average at the N/P ratio of 10, the cell viabilities of the four cholesterol-peptides followed an increasing order as Ch-R5H5 (59%)>Ch-R3H3 (64%)>Ch-R5 (72%)>Ch-R3 (76%), which were similar or slightly higher than that of PEI (70%) (Fig. 5A). In MCF-7 cells, a decreasing trend in cell viability was observed in all the cholesterol-peptide/DNA complexes as the N/P ratio was increased from 1 to 30. Above the N/P ratio of 5, at which a higher luciferase expression was achieved, the percentage of viable cells was comparable or lower than that of PEI at its optimal N/P ratio of 10. Among four cholesterol-peptide/DNA complexes, Ch-R5H5/DNA complexes showed a slightly higher cytotoxicity than the others, at the same N/P ratio ranging from 5 to 30. In general, the cell viability of MCF-7 cells treated with naked DNA, PEI/DNA and cholesterol-peptide/DNA complexes were lower than that of HEK-293 cells. The difference might be explained by that MCF-7 cells were more sensitive to foreign materials than HEK-293 cells. Cellular cytotoxicity may also be attributed to high zeta potential of cholesterol-peptide/DNA complexes since higher zeta potential of particles could cause membrane disruption [29].

### Conclusions

In this study, amphiphilic cholesterol-conjugated peptides were synthesized and investigated as gene delivery vectors. The amphiphilic cholesterol-peptides were able to self-assemble into cationic micelles at relative low concentrations (≤85 µg/mL), compared with hydrophilic cationic oligoarginine (R10). The micelles present in the solution increased local cationic-charge density and thus condensed DNA more efficiently, leading to high gene expression in both HEK-293 and MCF-7 cells. Moreover, by introducing histidine residues into cholesterol-peptides (Ch-R5H5 and Ch-R3H3), the overall gene expression level was increased compared with that of cholesterol-peptides without histidine (Ch-R5 and Ch-R3). Ch-R5H5 mediated great gene transfection efficiency. Also, the size and zeta potential of Ch-R5H5/DNA complexes (180 nm, 23 mV) was favorable for intravenous therapy. The results indicated that Ch-R5H5 is a promising candidate as gene delivery carrier for us to further study and optimize.
